# Comparison of Effectiveness of Gefitinib, Erlotinib, and Afatinib in Advanced Non-small Cell Lung Cancer Patients with *EGFR* Mutation Positive in Indonesian Population

**DOI:** 10.3779/j.issn.1009-3419.2019.09.02

**Published:** 2019-09-20

**Authors:** Noorwati SUTANDYO, Arif HANAFI, Mulawarman JAYUSMAN

**Affiliations:** 1 Division of Hematology and Medical Oncology, Department of Internal Medicine; 2 Department of Pulmonology, Dharmais National Cancer Centre Hospital, Jakarta, Indonesia

**Keywords:** Key words Lung neoplasms, *EGFR* mutation positive, Tyrosine kinase inhibitors

## Abstract

**Background and objective:**

EGFR-tyrosine kinase inhibitors (EGFR-TKIs) were used to treat non-small cell lung cancer (NSCLC) patients with *EGFR* mutation positive. This study aims to compare the effectiveness of first line TKIs; gefitinib, erlotinib, and afatinib in the treatment of advanced stage NSCLC patients with *EGFR* mutation positive in the Indonesian population.

**Methods:**

A retrospective cohort study of 88 NSCLC patients with *EGFR* mutation positive treated with gefitinib (*n*=59), erlotinib (*n*=22), and afatinib (*n*=7) was performed in national cancer hospital in Indonesia.Inclusion criteria were stage Ⅲb or Ⅳ NSCLC with adenocarcinoma subtype. Subjects less than 18 years or with a history of other malignancy were excluded. Outcomes were treatment response, progression-free survival (PFS), and mortality rate.

**Results:**

Complete response, partial response, and stable disease were shown in 1.1%, 35.2%, and 31.8% of subjects, respectively. There were 31.8% of subjects developed progressive disease during treatment. Regarding *EGFR* mutation positive profile, a total of 56.8% subjects had deletion in exon 19, 42% subjects had mutation in exon 21, and rare mutation in exon 18 was found in 3.4% of total subjects. Demography and clinical characteristics had no significant association with the risk of progressive disease. The median PFS of subjects was 11 months (95%CI: 6.8-15.2 months). There was no statistical difference of PFS between treatment groups.

**Conclusion:**

Gefitinib, erlotinib, and afatinib have similar effectiveness in advanced stage NSCLC with *EGFR* mutation positive. Afatinib tends to be associated with longer PFS but further investigation is required.

## Introduction

Despite the global health effort on smoking cessation, lung cancer still retains its high mortality rate in the developed and developing countries until present^[1]^. It was estimated that lung cancer mortality in 2035 will be 86% higher than in 2012^[2]^. Throughout all types of lung cancer cases, the non-small cell lung cancer (NSCLC) accounts for 85% of them. Adenocarcinoma subtype found in more than 70% of NSCLC. In a majority of patients, NSCLC is usually diagnosed at an advanced stage where surgical therapy is no longer applicable^[3]^.

In 10%-35% of lung adenocarcinoma, mutations in the epidermal growth factor receptor (*EGFR*) gene was found^[4]^. *EGFR* mutations were found in significantly higher proportion in female patients, Asian population, and non-smokers. The most common mutations were the deletion in exon 19 and mutation in exon 21 L858R point^[5]^. Several experimental studies and *meta*-analysis reported that EGFR-tyrosine kinase inhibitors (EGFR-TKIs) treatment has better efficacy in advanced stage of NSCLC with these *EGFR* mutation positive compared with conventional chemotherapy treatment^[6-9]^.

Currently, there are several EGFR-TKIs treatment such as gefitinib, erlotinib, afatinib worldwide approved for treating advance stage of NSCLC with *EGFR* mutation positive. Gefitinib and erlotinib are an oral reversible first-generation EGFR-TKIs. They bind to the ATP-binding sites to block the activation of the signal induced by EGFR. While afatinib is an oral irreversible second-generation EGFR-TKI. This drug was developed in response to the resistance of the first generations^[10]^.

However, several studies comparing the efficacy of gefitinib, erlotinib, and afatinib in lung adenocarcinoma patients' mortality and progression-free survival showed conflicting results^[11-15]^. In addition, there were only a limited number of similar studies in the South-East Asian population which possibly having different characteristics of *EGFR* mutations compared to East Asian, European, and American populations. So we conducted this study to compare the effectiveness of gefitinib, erlotinib, and afatinib in advance stage adenocarcinoma NSCLC patients with *EGFR* mutations in the Indonesian population.

## Methods

### Study design and population

This was a retrospective cohort study at Dharmais National Cancer Hospital, Indonesia. The study was approved by the Ethical Committee of Dharmais National Cancer Hospital. To optimize the power of the research, total sampling was performed in recruiting study subjects. Subjects were advanced non-small cell lung cancer (NSCLC) patients (adenocarcinoma subtype) with proven *EGFR* mutation positive, who were administered with gefitinib, erlotinib, or afatinib in the period of January 2013 to March 2015. *EGFR* mutations were analyzed in the Kalbe Genomic biomolecular laboratory, Indonesia. DNA was extracted from tumor tissue during the diagnostic procedure using the QIAamp blood kit (Qiagen, Hilden, Germany). Then, DNA amplification using high-resolution PCR protocol followed by direct DNA sequencing was performed to determine the *EGFR* mutation profile.

Inclusion criteria were stage Ⅲb or Ⅳ of lung adenocarcinoma according to American Joint Committee 2010.^[16]^ Subjects less than 18 years or with a history of other malignancy were excluded. EGFR-TKIs treatment was administered orally with a daily dose of 250 mg for gefitinib, 150 mg for erlotinib, or 40 mg for afatinib. Treatment would be discontinued if there was evidence of progressive disease or serious adverse event.

The demographic and clinical parameters were collected before the EGFR-TKIs treatment. These data included age, gender, body mass index (BMI), comorbidity, *EGFR* mutation status. We did a 60-month follow-up through the medical record to evaluate treatment response, progression-free survival (PFS), and mortality rate. Treatment response was assessed on the basis of the Response Evaluation Criteria in Solid Tumors (RECIST) guideline.^[17]^ Evaluation of the treatment response was performed every 3-6 cycles after starting EGFR-TKIs treatment. Clinical examination, laboratory tests, abdominal ultrasonography, and computed tomography (CT) scan were performed to determine treatment response.

### Statistical analysis

Statistical analysis was performed using IBM SPSS software version 24. Study outcomes were treatment response and 24-months PFS. For the survival analysis, we performed right censoring for handling loss to follow-up subjects.

To assess the association between type of EGFR-TKIs treatment and study outcomes, the *Chi-square* test was performed. A *P*-value of less than 0.05 was considered statistically significant. Relative risks and their 95% confidence interval were calculated.

The *Kaplan-Meier* graph and *log-rank* test were performed to compare the survival probability of PFS regarding EGFR-TKIs treatment.

## Results

A total of 115 of NSCLC patients fulfilled inclusion criteria of study. However, 27 subjects had incomplete medical records, so a total of 88 subjects were included in analysis.

## Subjects' Characteristics

Characteristics of subjects treated with gefitinib, erlotinib, and afatinib were comparable with respect to the demographic, clinical, and molecular variables (Table 1).

**1:** Characteristics of subjects [*n* (%)]

Characteristics	Total (*n*=88)	Gefitinib (*n*=59)	Erlotinib (*n*=22)	Afatinib (*n*=7)
Age				
Age (Mean±SD, yr)	60±11	60±12	59±9	56±9
> 60	47 (53.4)	31 (52.5)	12 (54.5)	4 (57.1)
< 60	41 (46.6)	28 (47.5)	10 (45.5)	3 (42.9)
Gender				
Male	47 (53.4)	30 (50.8)	14 (63.6)	3 (42.9)
Female	41 (46.6)	29 (49.2)	8 (36.4)	4 (57.1)
BMI (Mean±SD, kg/m^2^)	22.4±3.1	22.3±3.2	22.5±3.3	22.4±3.0
Stage of disease				
Stage Ⅲ	14 (15.9)	10 (16.9)	4 (18.2)	0 (0.0)
Stage Ⅳ	74 (84.1)	49 (83.1)	18 (81.8)	7 (100.0)
Presence of comorbidity				
Yes	24 (27.3)	13 (22.0)	10 (45.5)	1 (14.3)
No	64 (72.7)	46 (78.0)	12 (54.5)	6 (85.7)
Charlson comorbidity index				
> 5	72 (82.8)	48 (82.8)	17 (77.3)	7 (100.0)
≤5	15 (17.2)	10 (17.2)	5 (22.7)	0 (0.0)
*EGFR* mutation status^1^				
Deletion exon 19	50 (56.8)	31 (52.5)	14 (63.8)	5 (71.4)
Mutation exon 21	37 (42.0)	27 (45.8)	9 (40.9)	1 (14.4)
Mutation exon 18	3 (3.4)	2 (3.4)	0(0.0)	1 (14.3)
^1^Not mutually exclusive; BMI: body mass index; EGFR: epidermal growth factor receptor.				

The mean age of all subjects was 60 years. There was no significant difference in gender proportion. Most subjects were at stage Ⅳ. The most common sites of metastasis were pleura (51.4%), bone (31.1%), and brain (10.8%). More than 50% of total subjects were found to have exon 19 deletion in *EGFR* gene. Approximately 3% of total subjects had rare mutation in the *EGFR* gene (mutation in exon 18). There were two subjects having double mutations in exon 19 and exon 21.

## Response rate

Complete response (CR), partial response (PR), and stable disease (SD) were shown in 1.1%, 35.2%, and 31.8% of subjects, respectively. However, there were 31.8% of subjects who developed progressive disease during treatment of TKIs. Demography and clinical characteristics had no significant association with the risk of progressive disease (Table 2).

**2:** Association of demographic and clinical characteristics with response to EGFR-TKIs treatment [*n* (%)]

Variables	PD (*n*=28)	CR/PR/SD (*n*=60)	*P*	RR (95%CI)
Age (yr)				
≥60	12 (42.9)	35 (58.3)	0.175	0.82 (0.61-1.10)
< 60	16 (57.1)	25 (41.7)	1.000	Reference
Gender				
Female	9 (32.1)	32 (53.3)	0.063	0.76 (0.57-1.02)
Male	19 (67.9)	28 (46.7)	1.000	Reference
BMI (Mean±SD, kg/m^2^)	22.8±3.4	22.2±3.0	0.430	-0.57 (-2.09-0.95)1
Stage of disease				
Stage Ⅲ	4 (14.3)	10 (16.7)	1.000	Reference
Stage Ⅳ	24 (85.7)	50 (83.3)	0.776	1.06 (0.73-1.53)
EGFR-TKIs treatment				
Gefitinib	18 (64.3)	41 (68.3)	1.000	Reference
Erlotinib	7 (25.0)	15 (25.0)	1.000	1.04 (0.51-2.15)
Afatinib	3 (10.7)	4 (6.7)		1.40 (0.55-3.59)
Presence of comorbidity				
Yes	10 (35.7)	14 (23.3)	0.224	1.23 (0.85-1.79)
No	18 (64.3)	46 (76.7)	1.000	Reference
CCI				
> 5	24 (85.7)	49 (81.7)	0.638	1.09 (0.77-1.54)
≤5	4 (14.3)	11 (18.3)	1, 000	Reference
*EGFR* mutations				
Common mutation (in exon 19 or exon 21)	27 (96.4)	58 (96.7)	1, 000	Reference
Uncommon mutation	1 (3.6)	2 (3.3)	0.954	1.02 (0.45-2.31)
CR: complete response; PR: partial response; SD: stable disease; PD: progressive disease; 1Mean difference (95% CI of mean difference)				

Regarding the type of EGFR-TKIs treatment, the risk of progressive disease in subjects receiving gefitinib, erlotinib, and afatinib was similar. Subjects with uncommon *EGFR* mutations had no significantly difference of risk of progressive disease than subjects with a deletion in exon 19 or mutation in exon 21.

## Progression-free survival

The median progression-free survival (PFS) of subjects was 11 months (95%CI: 6.8-15.2 months). Comparison of 24 months PFS by EGFR-TKIs treatment is shown in figure 1. The median PFS of subjects receiving gefitinib and erlotinib were 9 and 13 months, respectively. While subjects receiving afatinib did not reach median survival in a 24-months follow-up. *Log-rank* test showed that there was no significant difference in PFS between these three groups of treatment (*P*=0.28).

**1:**
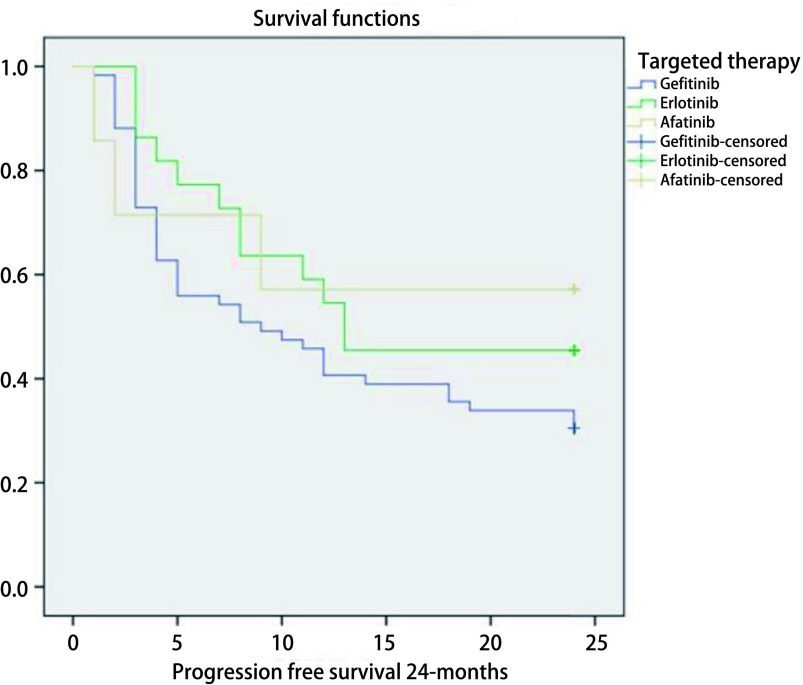
*Kaplan-Meier* graph of progression-free survival by EGFR-TKIs treatment group. TKI: tyrosine kinase inhibitors.

## Discussion

To our knowledge, this was the first Indonesian cohort study which compared the effectiveness of EGFR-TKIs treatment in advanced stage NSCLC patients with respect of *EGFR* mutation profile. The results of our study showed that gefitinib, erlotinib, and afatinib had similar effectiveness in terms of treatment response and 24-months follow-up of PFS. Although not statistically significant, subjects receiving afatinib have marginally longer PFS compared to others receiving gefitinib or erlotinib. No progression found in patients with afatinib in 24 months follow up. Regarding *EGFR* mutation status, subjects with uncommon *EGFR* mutations had similar risk of progressive disease compared to subjects with mutation in exon 19 or exon 21 point.

A retrospective study by Krawczyk *et al*.^[11]^ showed similar results. The study was done in Poland involving 180 NSCLC patients. They found that there was no significant in treatment response, PFS, and overall survival in NSCLC patients treated with gefitinib, erlotinib, and afatinib. There were 14.5% of subjects having progressive disease, compared to 31.8% in our study. The difference was probably due to several reasons. First, the study enrolled all subtypes of NSCLC in the Caucasian population compared to adenocarcinoma patients of the Asian population in our study. Second, there was a higher proportion of exon 19 mutation and a lower proportion of exon 21 in that study compared to ours. Patients with exon 19 deletion indicated to have a higher response rate after EGFR-TKI treatment compared patients having exon 21 mutation^[18, 19]^.

Similar to our study, Krawczyk *et al*.^[20]^ also reported that subjects treated with afatinib have slightly longer PFS compared to subjects receiving gefitinib or erlotinib. A large phase 2B randomized controlled trial comparing the efficacy of gefitinib and afatinib performed by Park *et al*. (Lung-LUX 7) supports this finding. The study showed that median PFS in the afatinib group was 11 months (95%CI: 10.6-12.9), while in gefitinib group was 10.9 months (95%CI: 9.1-11.5). However, it is important to note that there was a slightly higher incidence of serious treatment-related adverse event and fatal adverse event in afatinib group compared to gefitinib group (11% in afatinib group *vs* 4% in gefitinib group, 9% in afatinib group *vs* 6% in gefitinib group, respectively).

On the contrary, a large retrospective cohort study involving 7, 222 lung adenocarcinoma patients in Taiwan by Chang *et al* found a different result compares to our study^[21]^. Gefinitib showed superior efficacy compared to erlotinib. Subjects treated with gefinitib have longer PFS and overall survival in 1-year follow up. These differences could be due to several explanations. First, the erlotinib group in the study has a higher proportion of cachexia which could affect treatment response and overall survival. Second, there was no data on the type of *EGFR* mutations. So, there might be a possibility that there was a difference in the profile of *EGFR* mutation compared to our study population.

In our study, the multivariate analysis to control all of the potential confounders was not performed due to two considerations. First, despite total sampling, the sample size was not adequate to perform a multivariate analysis. Second, the baseline characteristics between gefitinib, erlotinib, and afatinib group were comparable. Moreover, other similar retrospective study performed by Krawcyzk *et al*.^[11]^ showed that patients' characteristics such as age, gender, staging, performance status, and smoking history did not affect one-year and two-year overall survival after multivariate cox regression analysis. Other retrospective cohort study performed by Chang *et al*.^[21]^ in Taiwan in previously treated NSCLC patients also reported that gender, comorbidities, and presence of cachexia had no association with PFS.

Our study had several limitations. Since afatinib were not covered in Indonesia National Health Insurance during the study period, we had only small number of subjects receiving afatinib. Therefore, although they had marginally longer PFS than other groups, the result must be interpreted cautiously. Further studies with larger sample size is needed to produce more precise result. Retrospective observational study designs was another limitation to our research. All potential confounders (*e.g.* smoking status, history of treatment) could not be analyzed due to incomplete data. In addition, time for assessing treatment response might be varied among study subjects.

## Conclusion

The result of this study provides evidence that gefitinib, erlotinib, and afatinib have similar effectiveness in advance stage *EGFR* mutation lung adenocarcinoma patients. Afatinib tends to associate with longer PFS but further investigation is required.
